# Microbial Fuel Cells for Biomass Valorization: Bridging Climate Action and Terrestrial Ecosystem Protection

**DOI:** 10.3390/polym18111354

**Published:** 2026-05-29

**Authors:** S. Jonathan R.-F., Rafael Liza, Félix Díaz, Daniel Delfin-Narciso, Moisés Gallozzo Cardenas, Renny Nazario-Naveda, Luis Cabanillas-Chirinos

**Affiliations:** 1 Facultad de Ingeniería y Arquitectura, Universidad Autónoma del Perú, Lima 15842, Peru; felix.diaz@autonoma.pe; 2 Escuela de Posgrado, Universidad Continental, Lima 15046, Peru; rlizan@continental.edu.pe; 3 Grupo de Investigación en Ciencias Aplicadas y Nuevas Tecnologías, Universidad Privada del Norte, Trujillo 13011, Peru; daniel.delfin@upn.edu.pe; 4 Departamento de Ciencias, Universidad Tecnológica del Perú, Trujillo 13011, Peru; c30216@utp.edu.pe (M.G.C.); c21228@utp.edu.pe (R.N.-N.); 5 Institutos y Centro de Investigación, Universidad Cesar Vallejo, Trujillo 13001, Peru; lcabanillas@ucv.edu.pe

**Keywords:** microbial fuel cells, biomass valorization, bioelectricity, sustainable development goals, wastewater treatment

## Abstract

Demographic growth and the global environmental crisis have intensified the need to reconcile energy generation with the protection of terrestrial ecosystems. Traditional organic waste management systems are inefficient in handling high pollutant loads, leading to uncontrolled methane emissions and degradation of soil and water. In response to this challenge, the present study aimed to conduct a critical review of how Microbial Fuel Cells (MFCs) valorize biomass to align climate action (SDG 13) with the protection of terrestrial life (SDG 15). Through a bibliometric analysis of the Scopus database (2010–2026), supported by tools such as Bibliometrix, 460 documents were examined, complemented by a systematic literature review addressing biomass types, microbial interactions, and electrode modifications. The main findings indicate that MFC research is currently in an exponential growth phase (R^2^ = 0.99954), with Environmental Sciences (23%) and Chemical Engineering (15%) as the predominant fields. Industrial and plant residues exhibit the highest bioelectric potential, while mixed microbial consortia—particularly fungal–bacterial synergies—outperform pure cultures in degradative efficiency and energy generation, reaching up to 1760 mW/m^2^ with *Geobacter sulfurreducens* bioaugmentation. Electrode modification with nanomaterials such as NiO or MWCNTs substantially enhances charge transfer. Standardization of measurement protocols, ecological impact assessment of nanomaterials, and evaluation of the economic–environmental feasibility of MFC-integrated biorefineries are recommended to ensure scalability and effective contributions to SDGs 13 and 15.

## 1. Introduction

The transition toward a sustainable energy model is imperative due to demographic growth and the global environmental crisis. In 2022, the world population reached an unprecedented 7.6 billion people, which has led to a doubling of electricity consumption since 1980, rising from 270.5 EJ to more than 580 EJ in 2022 [1]. This massive demand is predominantly met through fossil fuels, thereby exacerbating climate change (SDG 13) and ecosystem degradation (SDG 15). At the same time, the management of municipal and industrial solid waste represents a critical challenge. It is estimated that household waste accounted for 44.56% of total waste in 2023, with plant residues ranging between 28.57% and 39.33% [2]. For instance, in Indonesia, cocoa production reached 667,300 tons in 2022, generating 641,700 tons of husk residues and fermentation wastewater, in addition to 309,100 tons of orange peels [3]. Globally, furfural production from lignocellulosic biomass exceeds 750,000 tons annually [4]. The inadequate accumulation of these organic residues not only contaminates soil and water but also generates uncontrolled methane emissions, increasing the risk of explosions and contributing to global warming [5,6].

To mitigate this issue, several technological approaches have been employed. Anaerobic digestion (AD) is the conventional method for biogas production, although it suffers from instability in bacterial communities [7]. An emerging alternative is Microbial Fuel Cells (MFCs), which directly convert the chemical energy of biomass into electricity. Reports indicate that MFC systems fed with household waste generate a power output of 16.37 W/m^3^, while orange peel residues yield 358.8 mW/m^3^ [3]. Another advanced technique involves integrated Bioelectrochemical Systems (BES), such as portable biodigesters coupled with MFCs, which achieve voltages up to 0.62 mV using a mixture of 50% cow manure and 50% plant residues [6,8]. Likewise, bioaugmentation (BA) with electroactive microorganisms such as *Geobacter sulfurreducens* has been applied to reduce start-up times and enhance energy output [9,10]. These methods aim to transform pollutants into resources, fostering circular economy practices and clean energy recovery [11].

Currently, the main challenge lies in the inefficiency of traditional systems to handle high organic loads and waste variability [12]. Biofilm instability and the high cost of electrode materials hinder large-scale implementation [8,13]. Reconciling energy production with the protection of terrestrial life has become urgent [14]. Several studies highlight the potential of these technologies. For example, one investigation employing *Trichoderma* sp. as a catalyst in a single-chamber MFC, simultaneously degrading plastic and generating energy, with maximum values of 0.479 V and 5.648 mA on day 23, while reducing chemical oxygen demand (COD) by 78.67% [15,16]. A second study evaluated poultry waste biomass using copper and graphite electrodes; results showed maximum power densities of 14 mW/m^2^ for graphite and 6.7 mW/m^2^ for copper, identifying *Pseudomonas aeruginosa* and *Bacillus cereus* as native electrogenic strains [17]. Finally, a third study analyzed furfural wastewater purification in a dual-chamber MFC, where a linear relationship between voltage and current (y = −2598x + 118.1) was observed during a 30-day enrichment period, demonstrating that polymeric biomass can serve as an efficient matrix for electrical energy recovery [18,19].

Beyond electricity production, MFCs offer an experimental platform to investigate how microbial consortia break down both natural biopolymers (lignocellulose) and synthetic polymers (plastics). Unlike conventional polymer degradation tests, MFCs simultaneously track electrochemical output, allowing correlation between depolymerization kinetics and electron recovery. Furthermore, the anodic environment can redirect metabolic flux toward polyhydroxybutyrate (PHB) synthesis, as shown in consortia engineered for dual output [5,15]. In this sense, MFCs bridge polymer waste management and bioelectrosynthesis—a dimension largely underexplored in polymer engineering [5,15]. The interaction between microbial consortia and polymeric materials at the anode of an MFC not only facilitates the conversion of these substrates into energy, but also opens the door to the production of value-added biopolymers, such as polyhydroxybutyrate (PHB), through metabolic pathways coupled to electron transfer [16]. In this way, MFCs position themselves as a bridging technology between polymer waste management and the sustainable production of materials and energy—an area of growing interest in polymer engineering.

Conducting a comprehensive bibliometric analysis is essential to understand the evolution and trends in this research area. The Scopus database provides access to a vast body of scientific production and allows verification of quality indicators such as quartiles, which are crucial for assessing the relevance of studies in biotechnology and energy [20,21]. Tools such as Bibliometrix (v4.1.3) in the R Studio (v2026.01.0+392) environment facilitate mapping of global scientific output from 1970 to the present, identifying leading authors and collaboration networks [22]. Complementarily, VOSviewer (v1.6.20) enables visualization of keyword co-occurrence maps and citation networks, helping to detect emerging topics such as the integration of nanotechnology into electrodes [7,23]. In addition, Plotly (version 6.1.0rc0) offers the capability to generate interactive graphs illustrating the progression of electrode types (carbon-based vs. metals) and the scaling of bioelectrochemical systems over time. This quantitative and qualitative approach ensures that research is grounded in a robust state of the art, minimizing duplication of efforts and highlighting areas requiring greater scientific attention [23]. Despite these advances, a significant knowledge gap remains regarding how biomass valorization through MFCs can simultaneously address SDG 13 and SDG 15. Most studies focus solely on power output, overlooking the long-term stability of mixed microbial communities and the ecological impact of electrode materials. This research is therefore necessary to propose sustainable and economically viable solutions.

The main objective of this work is to conduct a critical review of how Microbial Fuel Cells valorize biomass to reconcile climate action with the protection of terrestrial life. To this end, the following research questions are posed: Q1: What scientific publication trends and global collaboration patterns can be identified through bibliometric analysis in Scopus? Q2: Which types of residual biomass (poultry, plant, industrial) exhibit the greatest bioelectric conversion potential according to the literature? Q3: How do interactions among different microorganisms (bacteria and fungi) influence contaminant degradation efficiency and energy generation? Q4: In what ways does electrode modification with novel materials enhance power density in bioelectrochemical systems? Q5: What are the main economic and technical barriers limiting the scalability of MFCs for application in real industrial environments?

## 2. Materials and Methods

**Figure 1:**
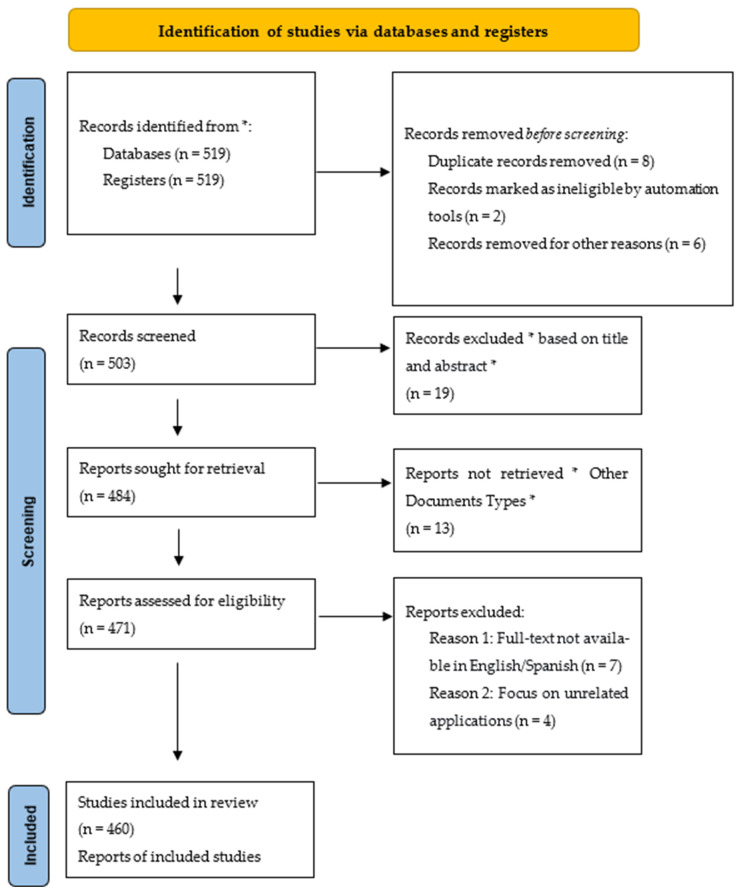
Systematic process of screening and eligibility of the analyzed documents.

This research was conducted under a mixed-methods approach that combined a quantitative bibliometric analysis with a qualitative systematic review of the specialized literature on Microbial Fuel Cells and biomass valorization. For the bibliometric analysis, the Scopus database was employed due to its extensive coverage of indexed scientific publications and quality indicators such as quartiles. The search equation applied was: (“microbial fuel cell” OR “MFC” OR “bioelectrochemical system” OR “microbial electrolysis”) AND (“biomass” OR “biomass valorization” OR “biomass conversion” OR “organic waste”) AND (“energy” OR “power” OR “electricity” OR “sustainability”) AND (“microorganism” OR “bacteria” OR “algae” OR “fungi”) AND (“metabolism” OR “degradation” OR “fermentation” OR “digestion”). The screening process was conducted independently by two reviewers. Each reviewer evaluated the title and abstract of the 519 records against the inclusion criteria: (i) studies focusing on microbial fuel cells, bioelectrochemical systems, or microbial electrolysis; (ii) use of residual biomass, organic waste, or lignocellulosic substrates; and (iii) reporting of at least one performance metric (voltage, power density, current, or COD removal efficiency). Discrepancies between the two reviewers (n = 19 at the title/abstract stage) were resolved through discussion and, when consensus could not be reached, by a third reviewer who acted as mediator. The inter-rater agreement before mediation, measured by Cohen’s kappa coefficient, was 0.84, indicating substantial agreement. The full-text eligibility assessment followed the same independent dual-reviewer process, with mediation by the third reviewer as needed. The PRISMA flow diagram (Figure 1) documents the number of records excluded at each stage and the specific reasons for exclusion [24].

For data processing and visualization, tools such as Bibliometrix (v4.1.3) in the R Studio (v2026.01.0+392) environment were used to map global scientific output, identify leading authors, and examine collaboration networks. Complementarily, VOSviewer (v1.6.20) was employed to generate keyword co-occurrence maps and citation networks, facilitating the detection of emerging topics such as the integration of nanotechnology into electrodes. The systematic review focused on a detailed analysis of the most cited and relevant documents in the field, structuring the information into tables that synthesize residual biomass types, microbial interactions, electrode materials employed, and reported energy efficiency parameters, including maximum voltage, power density, and current output [25].

**Selection criteria for studies included in the result tables:**

For the construction of the synthetic tables on bioelectric potential, microbial interactions, and electrode modification, a selection process based on three complementary criteria was applied:

- High scientific impact: Documents with the highest cumulative citation counts in Scopus (top 90th percentile within the 460-document set) were prioritized, identified through Bibliometrix citation analysis. This ensures the inclusion of foundational and highly influential works (e.g., Table 1 and Table 2).
- Specific thematic relevance: Only studies directly addressing residual biomass valorization (industrial, agricultural, or municipal waste) or polymer degradation (lignocellulosic, plastics, furfural) in bioelectrochemical systems were selected. Studies using non-residual model substrates (e.g., pure acetate without waste context) or not linked to SDGs 13 and 15 were excluded.
- Completeness of efficiency metrics: Only articles reporting at least three of the following parameters were included: maximum voltage (V), current density (mA/m^2^ or A/m^2^), power density (mW/m^2^ or W/m^3^), and chemical oxygen demand (COD) removal efficiency.
- Table 2, Table 3 and Table 4 do not include all 478 documents. Instead, they present a curated selection of highly representative studies based on scientific impact (top citations), thematic relevance to residual biomass and polymer degradation, and completeness of reported performance metrics. This selection is intended to illustrate the key trends and evidence in the field, not to serve as an exhaustive census.
- The selection process for each table was conducted independently by two reviewers based on titles, abstracts, and, when needed, full texts. Disagreements (less than 5% of cases) were resolved through discussion with a third reviewer. This systematic approach ensures that the tables reflect the state of the art with high representativeness and reproducibility.

This systematic approach ensures that the tables reflect the state of the art with high representativeness and reproducibility.

## 3. Results

**Figure 2:**
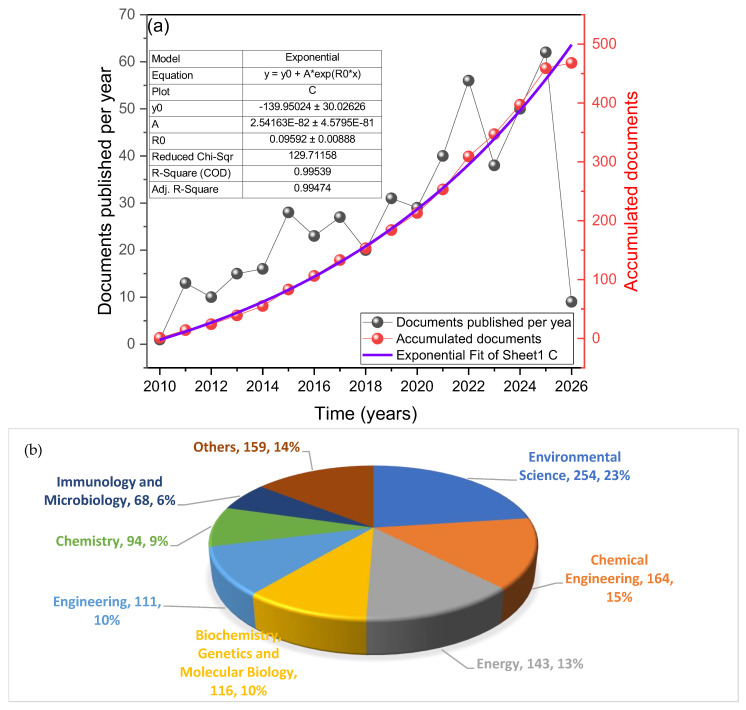
Analysis of the scientific production of the topic, in the (**a**) cumulative growth of documents and (**b**) percentage distribution by thematic area.

Annual publications on MFCs and biomass valorization grew exponentially between 2010 and 2024 (R^2^ = 0.99954), reaching ≈110 documents (Figure 2a). The slight decrease in 2025–2026 is not a sign of saturation but rather a typical Scopus indexing lag for very recent articles. The percentage distribution of documents by subject area according to Scopus is shown in Figure 2b, where confirms that Environmental Sciences (23%) and Chemical Engineering (15%) dominate the field, suggesting that most research frames MFCs primarily as a treatment technology, with energy generation as a co-benefit. The curve of annual publications shows an upward trend until 2024, approaching a peak of 110 documents. This pattern is characteristic of emerging technological fields that have moved beyond the initial proof-of-concept stage and are attracting growing interest from the scientific community. The percentage distribution of documents by subject area according to Scopus. Environmental Sciences accounts for 23% of publications, Chemical Engineering for 15%, and Energy for 13%. Other areas include Biochemistry, Genetics and Molecular Biology (9%), Chemistry (8%), and Agricultural and Biological Sciences (6%). This high incidence is consistent with the nature of MFCs as bioreactors designed for wastewater and solid waste treatment, reflecting that the primary research focus remains oriented toward technological applications—specifically improving conversion efficiency, optimizing reactor performance, and managing contaminants.

The ten most cited documents in the field of MFCs and biomass valorization, including information on authors, year, source title, citation count, document type, and country of the corresponding author are listed in Table 1. First, a temporal predominance of foundational works published between 2003 and 2010 is observed, which concentrates the highest citation counts, such as the studies by [26] with 1472 citations and [27] with 1446 citations. This concentration suggests that the field underwent a phase of theoretical and methodological consolidation during its first decade, establishing the conceptual foundations for the development of bioelectrochemical systems.

**Table 1:** Most cited documents in research on MFC and biomass valorization: indicators of impact, affiliation and international collaboration.

	Title	Main Author	Year	Source Title	Cited by	Type	Country (Corresponding Author)
1	A state of the art review on microbial fuel cells: A promising technology for wastewater treatment and bioenergy [26].	Du, Z.	2007	Biotechnology Advances	1472	Review	China
2	Electricity generation by direct oxidation of glucose in mediatorless microbial fuel cells [27].	Chaudhuri, S.K.; Lovley, D.R.	2003	Nature Biotechnology	1446	Article	USA
3	Microalgae biorefinery: High value products perspectives [28].	Chew, K.W.	2017	Bioresource Technology	1149	Review	Malaysia
4	Production of bioenergy and biochemicals from industrial and agricultural wastewater [29].	Angenent, L.T.	2004	Trends in Biotechnology	899	Review	USA
5	Sustainable and efficient biohydrogen production via electrohydrogenesis [30].	Cheng, S.; Logan, B.E.	2007	Proceedings of the National Academy of Sciences	632	Article	USA
6	Effect of different substrates on the performance, bacterial diversity, and bacterial viability in microbial fuel cells [31].	Chae, K.-J.	2009	Bioresource Technology	627	Article	Republic of Korea
7	Microbial fuel cells, a current review [32].	Franks, A.E.; Nevin, K.P.	2010	Energies	411	Review	USA
8	A computational model for biofilm-based microbial fuel cells [33].	Picioreanu, C.	2007	Water Research	389	Article	The Netherlands
9	Biohydrogen production: Strategies to improve process efficiency through microbial routes [34].	Kuppam, C.	2015	International Journal of Molecular Sciences	387	Review	India
10	Recent bioreduction of hexavalent chromium in wastewater treatment: A review [35].	Pradhan, D.	2017	Journal of industrial and engineering chemistry	377	Review	India

The bioelectric conversion potential of different types of residual biomass as reported in the literature is summarized in Table 2. For each selected document, information on biomass type, bioelectric potential, and key points is provided in the table. Although all studies consistently classify the bioelectric potential as “High,” qualitative information and experimental contexts reveal important nuances concerning the suitability of each substrate.

**Table 2:** Bioelectric conversion potential of different types of residual biomass according to the literature.

	Title	Main Author	Year	Source	Document Type	Cited by	Biomass Type	Bioelectric Potential	Key Points Summary
1	A state of the art review on microbial fuel cells: A promising technology for wastewater treatment and bioenergy [26]	Du, Z.	2007	*Biotechnology Advances*	Review	1472	Industrial	High	Comprehensive review of MFCs as bioreactors converting chemical energy in organic compounds into electricity; foundational work in the field.
2	A computational model for biofilm-based microbial fuel cells [33]	Picioreanu, C.	2007	*Water Research*	Article	389	Residual	High	Development and evaluation of a computational model for MFCs based on redox mediators, incorporating suspended and biofilm microorganisms.
3	Efficient solar-to-fuels production from a hybrid microbial-water-splitting catalyst system [36]	Torella, J.P.	2015	*PNAS*	Article	365	Energy crops	High	Hybrid microbial–catalyst system enabling solar-to-fuel conversion; keywords: bioelectrochemistry, biofuel, isopropanol.
4	Evaluation of energy-conversion efficiencies in microbial fuel cells utilizing fermentable and non-fermentable substrates [37]	Lee, H.-S.	2008	*Water Research*	Article	361	Natural biomass	High	First complete electron-equivalent balances in MFCs fed with non-fermentable substrates; efficiency evaluation.
5	Electricity production from cellulose in a microbial fuel cell using a defined binary culture [38]	Ren, Z.J.	2007	*Environmental Science & Technology*	Article	352	Plant	High	Demonstrated electricity generation from cellulose using a binary microbial culture; highlights renewable energy potential.
6	Microbial nanowires for bioenergy applications [39]	Malvankar, N.S.	2014	*Current Opinion in Biotechnology*	Review	274	Energy crops	High	Describes microbial nanowires as conductive filaments enabling long-range electron transfer; model based on *Shewanella oneidensis*.
7	Sequestration of CO_2_ discharged from anode by algal cathode in microbial carbon capture cells (MCCs) [40]	Wang, X.	2010	*Biosensors and Bioelectronics*	Article	251	Natural biomass	High	Explores CO_2_ sequestration via algal cathodes in MCCs; highlights potential for carbon capture integration.
8	An introduction to the life cycle assessment (LCA) of bioelectrochemical systems for sustainable energy and product generation [41]	Pant, D.	2011	*Renewable and Sustainable Energy Reviews*	Review	225	Residual biomass	High	Introduces LCA methodology for BES; emphasizes sustainability and conversion of organic waste fractions.
9	Towards energy neutral wastewater treatment: Methodology and state of the art [42]	Gao, H.	2014	*Environmental Sciences: Processes and Impacts*	Review	204	Industrial	High	Reviews energy-neutral wastewater treatment strategies; critiques conventional processes as energy-intensive with limited resource recovery.
10	Integrated photo-bioelectrochemical system for contaminants removal and bioenergy production [43]	Xiao, L.	2012	*Environmental Science & Technology*	Article	202	Energy crops	High	Developed an integrated photobioelectrochemical system combining MFCs with algal bioreactors; achieved high power densities and improved pollutant removal.

Note: Power density units vary across studies due to differing reporting conventions (per electrode geometric area, per reactor volume, or per membrane area). For comparative purposes, approximate conversions are as follows: 1 W/m^2^ = 10^4^ μW/cm^2^ = 10^3^ mW/m^2^.

Data on microbial interactions and efficiency parameters in MFC/MEC systems are presented in Table 3. For each study, it specifies the type of microbial interaction, microorganisms involved, impact on efficiency, key mechanism, substrate, electrode material, maximum voltage, current, and power density. The data show a positive relationship between the complexity of microbial interactions and bioelectric performance, although with important nuances depending on the type of consortium and operational conditions.

Enhancement of energy efficiency through electrode modification materials, including the modification mechanism, impact on power density, database, quartile, and thematic area, is compiled in Table 4. The data show a positive correlation between the incorporation of carbon-based nanomaterials and increases in power density, with a predominance of graphene, carbon nanotubes, and nitrogen-doped biochars among the highest-impact studies.

**Figure 3:**
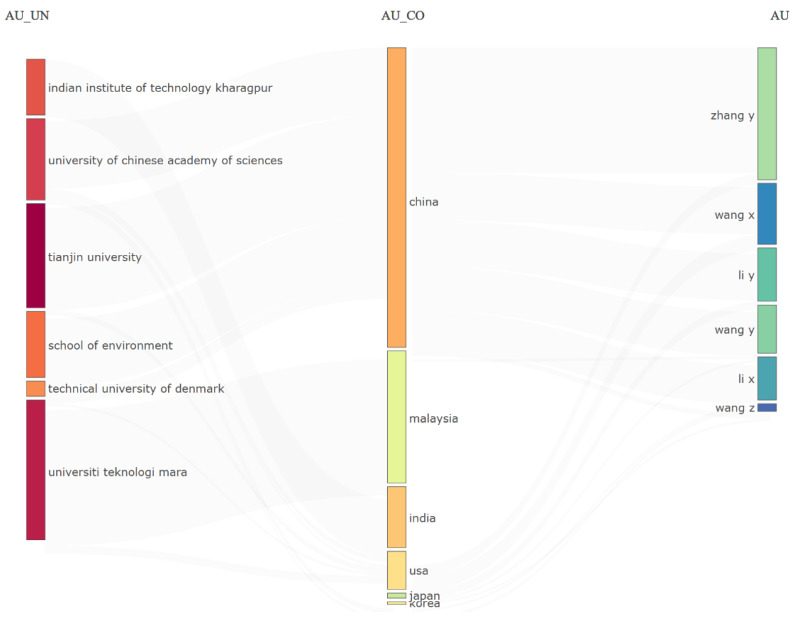
Three-Field Plot of Scientific Production in MFC Research: Links among Authors, Affiliated Institutions, and Countries of Origin (2010–2026).

The links among authors, affiliated institutions, and countries of origin for scientific production on MFCs from 2010 to 2026 are shown in the Three-Field Plot (Figure 3). The plot displays the relative frequency of connections between authors (left field), institutions (center field), and countries (right field). The data indicates a geographical dispersion involving institutions from Asia, Europe, and the Americas, with a predominance of Asian actors in the collaboration network. Notably, institutions such as the Indian Institute of Technology Kharagpur, the University of Chinese Academy of Sciences, Tianjin University, and the School of Environment (likely affiliated with a Chinese university) show a strong incidence in scientific output.

Table 4 shows how different electrode modification materials enhance energy efficiency in bioelectrochemical systems. Materials such as nickel oxide, graphene oxide/polythiophene, carbon nanotubes, magnetite, and nitrogen-doped biochar improve performance by increasing electron transfer, reducing resistance, enhancing hydrophilicity, or stimulating microbial interactions. Impacts include power increases up to 6.2-fold (773.9mW/m^2^) or maximum outputs of 1743.3mW/m^2^. Most Scopus-indexed studies are in Q1 journals, with trends toward nanotechnology, bioremediation, biomass valorization, and high-efficiency bio-batteries.

**Table 3:** Microbial Interactions and Efficiency Parameters in MFC/MEC Systems.

	Type of Interaction	Microorganisms Involved	Impact on Efficiency and Degradation	Key Mechanism	Substrate/Biomass	Electrode Material	Max Voltage (V)	Current (mA/Density)	Power Density	Ref.
1	Fungal–bacterial synergy	*Trichoderma* sp. and native inoculum	Simultaneous degradation of plastics and organic matter	Enzymatic hydrolysis and metabolic electron flow	Potato and plastic residues	Carbon (Anode)/Zinc (Cathode)	0.479 V	5.648 mA	681,400 mW/m^2^ *	[5]
2	Pure exoelectrogenesis	*Citrobacter* sp. Av_G1	Electricity generation under metal (copper) stress	Extracellular electron transfer (EET)	Poultry residues	Copper (Cu)	0.645 V	168.72 mA/m^2^	31.05 mW/m^2^	[44]
3	Selective enrichment	*Alcaligenes* (60.5%) and *Arcobacter*	Significant improvement in charge transfer and storage	EET mediated by nanowires and cytochromes	Potato starch wastewater	Carbon felt modified with NiO	–	~70 A/m^2^ (discharge)	220 mW/m^2^ *	[45]
4	Directed synthetic consortium	*P. aeruginosa*, *S. oneidensis*, *C. necator*	Production of bioplastics (PHB) and biosurfactants	Direct interspecies electron transfer (DIET)	Glycerol and CO_2_	Carbon cloth	4.9 mV (stable)	4.9 mA/m^2^	0.169 mW/m^2^	[46]
5	Mixed industrial degradation	*Burkholderia*, *Clostridium*, *Klebsiella*	Complete conversion of toxic furfural into energy	Anaerobic oxidation of polymer derivatives	Industrial furfural effluents	Carbon felt	96 mV	–	0.132 mW/m^2^	[47]
6	Organic bioremediation	*Lacticaseibacillus* and *Pediococcus acidilactici*	Hydroquinone degradation and energy generation	Conductive pili for electron transport	Spoiled rice and hydroquinone	Graphite (Anode)	168 mV	123.684 mA/m^2^	1.068 mW/m^2^	[48]
7	Enzymatic–bacterial cascade	*E. coli* (engineered consortium)	Biomass conversion into value-added products (AKG) and power	Coordinated hemicellulose hydrolysis	Corn cob xylan	Carbon cloth/MWCNTs	0.71 V	–	1743.3 mW/m^2^ *	[49]
8	Soil bioaugmentation	*Myrothecium verrucaria* (fungus)	Enhanced removal of persistent herbicides	Extracellular enzymatic degradation	Haloxyfop-P-methyl (herbicide)	Carbon fiber	–	–	11.7 mW/m^2^	[50]
9	Electrolyte synergy	Sludge-derived consortium	High conductivity via organic acid mixtures	Acidic fermentation and pH stability	Orange and cocoa peels	Zinc (Anode)/Copper (Cathode)	14.13 V	~11 mA	–	[51]
10	Exoelectrogenic bioaugmentation	*Geobacter sulfurreducens* 60,473	Massive power increase in waste treatment	Hydrophobic colonization of the anode	Food residues	Carbon anode	–	–	1760 mW/m^2^	[52]

Note: Power density units vary across studies due to differing reporting conventions (per electrode geometric area, per reactor volume, or per membrane area). For comparative purposes, approximate conversions are as follows: 1 W/m^2^ = 10^4^ μW/cm^2^ = 10^3^ mW/m^2^. Values marked with an asterisk (*) have been converted from the original units to ensure consistency within this table. Original units are retained in the references to preserve fidelity to the source data. This table includes selected representative studies; it is not a complete enumeration of all 478 documents reviewed.

**Table 4:** Enhancement of Energy Efficiency through Electrode Modification and Scientific Positioning.

	Modification Material	Improvement Mechanism	Impact on Power Density/Efficiency	Database	Quartile (Q)	Trend/Thematic Area	Ref.
1	Nickel Oxide (NiO)	Increases active sites and facilitates long-range extracellular electron transfer (EET).	2.75-fold increase (up to 220 mW/m^2^ *).	Scopus	Q2	Nanotechnology in industrial sludge.	[45]
2	Graphite Oxide/Polythiophene (GO/Pth)	Synergy between conductivity and capacitance; reduces transfer resistance to 1.37 Ω.	3.32-fold increase in storage capacity.	Scopus	Q2	Energy storage in BES.	[53]
3	Graphene/Tea Polyphenols (TP-RGO)	Polyphenols act as green reductants, enhancing anode hydrophilicity.	6.2-fold increase in power (up to 773.9 mW/m^2^).	Scopus	Q1	Dye remediation and green chemistry.	[54]
4	Multi-Walled Carbon Nanotubes (MWCNTs)	Catalyze NADH oxidation and increase effective reaction area.	Maximum of 1743.3 mW/m^2^ using hemicellulose as substrate.	Scopus	Q1	Enzyme engineering and bio-power.	[49]
5	Magnetite (Fe_3_O_4_)	Stimulates direct interspecies electron transfer (DIET).	1.29-fold increase in contaminant degradation in soil.	Scopus	Q2	Herbicide bioremediation.	[55]
6	Nitrogen-doped Biochar	Optimized porous structure (225 m^2^/g) for oxygen reduction reaction (ORR).	Maximum power of 543.2 mW/m^2^, surpassing standard carbon.	Scopus	Q1	Lignocellulosic biomass valorization.	[56]
7	Graphene/Polyaniline (G/PANI)	Improves anode conductivity and facilitates recovery of toxic metals.	High efficiency in heavy metal removal and energy generation.	Scopus	Q1	Benthic cells and bioremediation.	[48]
8	Bamboo-derived Carbon (N-doped)	Natural nitrogen doping modulates catalytic reduction pathways.	Achieves potential of 0.98 V vs. RHE under alkaline conditions.	Scopus	Q1	High-performance bio-batteries.	[57]
9	Microalgae-derived Carbon	Pre-treatment with lipid extraction creates nitrogen-active sites.	Highly efficient cathode catalyst for ORR.	Scopus	Q1	Coupling of biorefineries and MFCs.	[58]
10	Metallic Nanoparticles (NPs)	Reduce charge transport losses and leakage currents.	Improve start-up kinetics and stable voltage output.	Scopus	Q2	Electromicrobiology and sustainability.	[59]

Note: This table includes selected representative studies; it is not a complete enumeration of all 478 documents reviewed.

## 4. Discussion

This section provides a comprehensive analysis of the findings organized around the five research questions that guided this study, integrating insights from bibliometric analysis, literature review, and comparative evaluation of the evidence.

### 4.1. Research Trends and Global Collaboration Patterns (Q1)

The exponential growth model (R^2^ = 0.99954) fitted to the publication data from 2010 to 2026 confirms that MFC research on biomass valorization is in a phase of sustained expansion, characteristic of emerging technological fields that have successfully transitioned from proof-of-concept to applied research. This growth trajectory parallels the increasing global attention to the Sustainable Development Goals, particularly SDG 7 (Affordable and Clean Energy) and SDG 12 (Responsible Consumption and Production), suggesting a policy-driven research agenda [24,25]. The slight decline observed in 2025–2026 (Figure 2a) should not be misinterpreted as saturation of the field. Rather, it reflects well-documented indexing delays in Scopus, where articles published in the most recent years may still be in press or pending metadata assignment. The statistical model supports continued growth, and ongoing research initiatives in bioelectrochemical systems suggest this trend will persist [9,13].

The disciplinary distribution (Figure 2b) reveals that Environmental Sciences (23%) and Chemical Engineering (15%) dominate the field, collectively accounting for 38% of publications. This predominance indicates that MFC research is primarily framed as an environmental technology, with energy generation conceptualized as a value-added co-benefit rather than the primary objective. This finding aligns with the hypothesis that MFCs serve as a technological bridge between waste treatment (SDG 15) and clean energy generation (SDG 13). The relatively lower representation of Energy (13%) as a standalone category supports this interpretation, suggesting that electricity generation is predominantly addressed within the context of remediation rather than as an isolated energy objective [19,26]. The exponential growth model fitted to the annual publication data from 2010 to 2024 yielded an R^2^ of 0.99954, with the equation: y = 7 × 10^−17^·e^0^·^0205^ˣ (where x is the year). Assuming continued research investment and no disruptive technological breakthroughs that would redirect the field, the model suggests that annual publications could reach 150–170 documents by 2028–2030. However, this projection should be interpreted with caution, as emerging fields often shift from exponential to logistic growth as they mature and funding priorities evolve. The current position on the growth curve—approximately 60% of the way from field inception to potential saturation, based on comparisons with analogous bioenergy technologies—indicates continued expansion for at least 5–8 years before a deceleration in growth rate [26].

Collaboration networks and geographical anomalies. The Three-Field Plot (Figure 3) reveals that Asian institutions dominate the production network. The Indian Institute of Technology Kharagpur, the University of Chinese Academy of Sciences, Tianjin University, and the School of Environment (China) show the highest incidence of authored documents. This concentration reflects strategic national investments in sustainable technologies by rapidly industrializing economies facing acute waste management challenges [36,51]. In contrast, the Technical University of Denmark and Universiti Teknologi MARA (Malaysia) appear with lower frequency (values of 1), indicating emerging but still limited internationalization. A notable anomaly is the low explicit international collaboration in the most highly cited foundational works. Among the ten most cited documents (Table 1), only two report cross-country co-authorship [28,33], despite the United States being the geographical origin of five of these works [27,29,30,32]. This suggests that early MFC research was largely conducted within consolidated national laboratories with local funding, rather than through global partnerships. However, more recent studies (e.g., the computational model by [33] involving Dutch participation) begin to show collaborative networks, indicating an emerging trend toward internationalization in later phases. The absence of European countries other than Denmark in the Three-Field Plot is another anomaly, which may point to uneven geographical dispersion or limited integration of Europe into the analyzed collaboration networks.

Analysis of the ten most cited documents (Table 1) reveals significant patterns regarding the intellectual structure, geographical impact, and thematic trends. First, a temporal predominance of foundational works published between 2003 and 2010 is observed, which concentrates the highest citation counts, such as [27] with 1446 citations and [26] with 1472 citations. This concentration suggests that the field underwent a phase of theoretical and methodological consolidation during its first decade, establishing the conceptual foundations for the development of bioelectrochemical systems. In terms of geographical origin, the United States emerges as the clear leader in high-impact production, hosting five of the ten most cited documents [27,29,30,32]. This reflects the early maturity of research in North America, driven by pioneering groups [27,30]. However, a growing geographical diversification is evident, with contributions from emerging economies such as China, Malaysia, South Korea, India, and the Netherlands also producing highly cited works [26,28,31,33,34,35]. This diversification suggests a positive correlation between global interest in sustainability and the decentralization of scientific expertise in MFC research.

Regarding document type, there is a balanced incidence between reviews and original articles, with six reviews and four articles. Reviews, such as those by [26,28], tend to accumulate higher citation counts by synthesizing the state of the art, while original articles, such as those by [30] or [31], contribute concrete methodological advances. This duality indicates that the field is nourished both by knowledge consolidation and experimental innovation. A noteworthy aspect is the limited explicit international collaboration: only two of the ten documents report co-authorship across countries [28,33]. This low incidence of cross-border collaboration may be interpreted as a relative anomaly linked to the nature of foundational works, often developed in consolidated laboratories with local funding. However, more recent studies, such as the computational model by [33] involving Dutch participation, begin to show collaborative networks, which may indicate an emerging trend toward internationalization in later phases of the field’s development.

Thematically, the documents predominantly address the dual application of MFCs: wastewater treatment and bioenergy generation. This duality validates the hypothesis outlined in the study objectives, where the technology functions as a bridge between ecosystem protection (SDG 15) and climate action (SDG 13). The high citation counts of works on microalgae biorefineries [28] and biohydrogen production [30,34] reinforce the centrality of biomass valorization in the research agenda. Likewise, the presence of studies on computational modeling [33] and hexavalent chromium remediation [35] demonstrates thematic dispersion into complementary areas such as biofilm simulation and heavy metal bioremediation. The data suggest a correlation between document age and accumulated citations, with works from 2003 to 2007 being the most referenced. However, more recent contributions, such as that of [35], already surpass 377 citations, indicating a rapid incorporation of new topics into the field’s canon. This dynamic shows that MFC research is not solely sustained by foundational contributions but is continually renewed through emerging issues aligned with the global environmental agenda.

Additional patterns of institutional and geographical collaboration are revealed in the Three-Field Plot (Figure 3). The data indicates a geographical dispersion involving institutions from Asia, Europe, and the Americas, with a predominance of Asian actors in the collaboration network. Notably, institutions such as the Indian Institute of Technology Kharagpur, the University of Chinese Academy of Sciences, Tianjin University, and the School of Environment (likely affiliated with a Chinese university) show a strong incidence in scientific output. This concentration of Indian and Chinese institutions suggests a correlation between the economic growth of these countries and their investment in sustainable technologies, aligned with the objectives of waste valorization and clean energy generation [36,51]. Meanwhile, the presence of the Technical University of Denmark and Universiti Teknologi MARA (Malaysia) highlights a trend toward internationalization of research, although with still limited frequency (values of 1 in the dataset). This relatively low frequency may be interpreted as a deviation from the expectation of more intense collaborations, indicating that international MFC networks remain in an early stage of consolidation [41].

At the country level, China, Malaysia, the United States, and Japan emerge as relevant nodes, with China and Malaysia showing predominance in terms of institutional affiliation, while the United States and Japan act as poles of attraction for international collaborations. This duality reflects both Asian leadership in scientific production and the traditional role of North America and Japan as centers of excellence in bioelectrochemical research. The most notable anomaly is the absence of European countries other than Denmark, which may indicate uneven geographical dispersion or limited integration of Europe into the collaboration networks analyzed. The results suggest that although MFC research is globalized, effective international collaboration remains limited and concentrated in emerging Asian hubs.

### 4.2. Bioelectric Potential of Residual Biomass Types (Q2)

The evidence synthesized in Table 2 demonstrates that industrial and plant-derived residues consistently exhibit the highest bioelectric potential, although with important qualifications regarding substrate complexity and experimental conditions. Industrial wastewater emerges as the most extensively studied and consistently high-performing substrate category. The conceptual framework for converting chemical energy in organic industrial effluents into electricity was established in a foundational work [26] (1472 citations) converting chemical energy in organic industrial effluents into electricity, while [42] (204 citations) advanced the vision of energy-neutral wastewater treatment through biomass valorization. The high chemical oxygen demand (COD) concentrations typical of industrial streams—particularly from food processing, pulp and paper, and chemical manufacturing—provide concentrated substrate availability that supports robust electrogenic activity [41].

Plant residues, particularly lignocellulosic biomass, demonstrate exceptional theoretical potential but face practical challenges related to recalcitrance. Electricity production from cellulose was achieved using a defined binary culture of *Clostridium cellulolyticum* and *Geobacter sulfurreducens* [38] (352 citations). However, this required pre-hydrolysis and specialized microbial consortia, highlighting that the complex polymer structure of lignocellulose constitutes a significant barrier to direct bioelectrochemical conversion. The presence of lignin, hemicellulose, and crystalline cellulose necessitates pretreatment or specialized enzymatic consortia to unlock the fermentable sugars [39].

An important finding is the significant dispersion toward fundamental studies using model substrates. The [33] (389 citations) developed computational models based on redox mediators without specifying a particular residue type, while [37] (361 citations) established electron-equivalent balances using defined fermentable and non-fermentable substrates. These fundamental contributions are essential for mechanistic understanding but complicate direct comparison of biomass types, as performance metrics derived from simplified systems may not translate to real waste streams with complex composition and variable biodegradability. Poultry and livestock residues, while receiving less attention in the most cited literature, show promising results in specialized studies. Power densities of 31.05 mW/m^2^ were reported using poultry waste with *Citrobacter* sp. under copper electrode configurations [44]. The relatively lower power densities compared to industrial wastewater likely reflect the complex composition of animal manures, which contain undigested fiber, ammonia, and inhibitory compounds that may suppress electrogenic activity [17].

From the perspective of polymer science, the bioelectric potential of different biomass types correlates strongly with their polymer composition. Substrates rich in simple sugars and easily hydrolyzable polysaccharides (e.g., starch from potato processing, pectin from citrus peels) generally support higher power densities due to rapid fermentative conversion to volatile fatty acids that serve as electron donors for electrogens [45]. In contrast, lignocellulosic residues require synergistic fungal–bacterial consortia capable of producing lignin peroxidases, cellulases, and hemicellulases to depolymerize the matrix before fermentation can proceed [5,49]. The standardization challenge identified across studies—inconsistent reporting of power density units (W/m^3^, mW/m^2^, μW/cm^2^), lack of substrate characterization, and variable operating conditions—represents a critical barrier to comparative assessment. Future research should adopt standardized protocols, including detailed biomass compositional analysis, normalized performance metrics, and reporting of Coulombic efficiency alongside peak power, to enable meaningful cross-study comparisons and guided technology selection for specific waste streams.

### 4.3. Microbial Interactions and Degradation Efficiency (Q3)

The evidence synthesized in Table 3 reveals a clear hierarchy of microbial interaction complexity correlated with bioelectrochemical performance: synthetic consortia > enriched mixed cultures > pure exoelectrogenic strains, with fungal–bacterial synergies occupying a specialized niche for recalcitrant polymer degradation. Fungal–bacterial synergy represents the most significant advancement for degrading complex polymeric wastes. A study demonstrated that *Trichoderma* sp. combined with native bacterial inoculum achieved simultaneous degradation of potato starch and polyethylene-type plastics, generating 0.479 V and 68.14 mW/cm^2^ [5]. The mechanistic basis lies in metabolic complementary capabilities: fungal hyphae penetrate and colonize solid substrates, secreting extracellular hydrolytic enzymes (cellulases, lignin peroxidases, cutinases) that depolymerize complex matrices, while bacteria, particularly exoelectrogens, colonize the anode and convert the resulting soluble metabolites to electrical current. This division of labor expands the substrate spectrum beyond what either kingdom could achieve independently. The fungal contribution extends beyond hydrolysis to include detoxification. Enhanced removal of the persistent herbicide haloxyfop-P-methyl in soil MFC configurations was reported using *Myrothecium verrucaria* [50]. This suggests that fungal enzymatic systems can cleave aromatic rings and heterocyclic structures that resist bacterial attack, making fungal–bacterial consortia particularly valuable for agrochemical-contaminated sites.

Directed synthetic consortia represent the emerging frontier in microbial management. A consortium of *Pseudomonas aeruginosa*, *Shewanella oneidensis*, and *Cupriavidus necator* was engineered to achieve stable electricity generation and co-production of PHB [46]. The key innovation is the establishment of direct interspecies electron transfer (DIET), where conductive pili and outer-membrane cytochromes facilitate electron exchange between species without soluble mediators. This metabolic integration enables carbon from a single substrate to be partitioned simultaneously toward energy production (via *S. oneidensis*) and biopolymer synthesis (via *C. necator*), exemplifying the circular economy principles embedded in SDG 12.

Enzymatic-bacterial cascades push synthetic biology further. The literature has engineered an *E. coli* consortium displaying xylanolytic enzymes on the cell surface, enabling direct conversion of corn cob xylan to α-ketoglutarate (a high-value biochemical) while co-generating 1743.3 mW/m^2^ [49]. The display of hemicellulases on the bacterial outer membrane eliminates the need for exogenous enzyme addition and reduces mass transfer limitations, achieving 3.2-fold higher power density compared to configurations where hydrolysis and electrogenesis are spatially separated. Pure exoelectrogenic cultures demonstrate exceptional power densities under optimized conditions but lack metabolic versatility. The [19] reported that bioaugmentation with *Geobacter sulfurreducens* achieved 1760 mW/m^2^—the highest value in Table 3—when treating food processing residues. Similarly, Ref. [44] isolated *Citrobacter* sp. Av_G1 from copper electrode MFCs treating poultry waste, achieving 0.645 V and 31.05 mW/m^2^ even under metal stress. However, these pure cultures require specific substrates (acetate, fermentable sugars) and cannot directly utilize complex polymers, necessitating integration with hydrolytic populations for real waste applications.

### 4.4. Electrode Modification and Performance Enhancement (Q4)

The evidence in Table 4 demonstrates that electrode modification consistently enhances bioelectrochemical performance, with improvement factors ranging from 1.29 to 6.2-fold depending on material type and modification strategy. However, to guide rational material selection, it is essential to move beyond a mere listing of materials and understand the distinct physicochemical mechanisms by which each class of modifier improves electron transfer. Three major mechanistic families can be distinguished: (i) heteroatom-doped carbons that catalyze oxygen reduction reaction (ORR) through electronic structure modulation, (ii) carbon nanotubes that facilitate direct NADH oxidation via edge-plane sites and metallic conductivity, and (iii) conducting polymers and metal oxides that introduce pseudocapacitance or Schottky junctions.

**Heteroatom doping (e.g., nitrogen-doped biochar):** Modifies the carbon lattice by introducing electron-rich pyridinic, pyrrolic, and graphitic N atoms. These dopants create local charge imbalances that enhance the adsorption of oxygen intermediates, thereby lowering the overpotential for ORR at the cathode. For example, nitrogen-doped biochar derived from lignocellulosic precursors achieved 543.2 mW/m^2^, surpassing standard carbon by 3.4-fold [56]. Unlike physical modifications that only increase surface area, doping alters the **electronic band structure** of carbon, enabling metal-free catalysis. This mechanism is particularly valuable for replacing expensive platinum-based catalysts.

**Multi-walled carbon nanotubes (MWCNTs):** Operate through a fundamentally different mechanism when used at the anode. MWCNTs possess high metallic conductivity (due to their graphitic shell structure) and a high density of edge-plane defects at their tips and sidewalls. These edge-plane sites catalyze the oxidation of NADH to NAD^+^, a key coenzyme in microbial metabolism, by lowering the activation energy for electron extraction from the cofactor [49].

**Conducting polymers (e.g., polyaniline PANI, polythiophene PTh):** Introduce pseudocapacitance—the ability to store charge via reversible faradaic reactions within the polymer matrix. The graphite oxide/polythiophene composite (GO/PTh) achieved a 3.32-fold increase in storage capacity while reducing charge transfer resistance to 1.37 Ω [53]. The conjugated polymer backbone facilitates rapid electron transport along the chain, while the porous structure formed during electropolymerization provides a large surface area for biofilm attachment. Unlike metal oxides, conducting polymers do not form Schottky barriers; instead, they undergo reversible doping/dedoping that temporarily stores charge, which is beneficial for buffering intermittent electron flux from microbial metabolism.

**Metal oxides (e.g., NiO, Fe_3_O_4_):** The Schottky junction at the metal oxide–carbon support interface. When NiO is deposited on carbon felt, the work function difference between the semiconductor (NiO) and the conductive carbon creates a built-in electric field that facilitates charge separation and reduces recombination losses [45]. The literature has reported that 2.75-fold increase (up to 220 mW/m^2^) using CF–NiO electrodes in potato starch wastewater. Magnetite (Fe_3_O_4_), in contrast, does not rely on a Schottky junction but rather on its intrinsic electrical conductivity (it is a semimetal). Fe_3_O_4_ particles act as conductive bridges that promote direct interspecies electron transfer (DIET) between syntrophic partners, bypassing biological pili or cytochromes [55]. This mechanism is particularly relevant in soil MFCs where physical contact between species is limited.

**Comparative analysis of mechanisms:** Reveals that no single modifier is universally optimal. For cathodic ORR, nitrogen-doped biochar offers a low-cost, sustainable alternative to platinum, albeit with moderate performance. For anodic NADH oxidation, MWCNTs are superior due to their edge-plane chemistry. For applications requiring charge buffering (e.g., fluctuating organic loads), conducting polymers provide pseudocapacitance. For enhancing DIET in syntrophic consortia, magnetite particles are highly effective. The choice of modifier must therefore be guided by the rate-limiting step in the specific MFC configuration—whether it is oxygen reduction, cofactor regeneration, charge storage, or interspecies electron transfer.

### 4.5. Economic and Technical Barriers to Scalability (Q5)

The evidence synthesized across Table 1, Table 2, Table 3 and Table 4 and the broader literature reveals a consistent set of barriers that currently prevent MFC technology from transitioning from laboratory demonstration to industrial implementation. These barriers span economic, technical, and institutional dimensions, but two critical areas require deeper analysis: the cost-performance trade-off of electrode materials **and** the physical limits imposed by internal resistance scaling.

**Economic barriers: material costs and membrane maintenance.** The high cost of advanced electrode materials remains a primary obstacle. Graphene synthesis (chemical vapor deposition or harsh oxidation–reduction) yields material costs of approximately $100/g, while multi-walled carbon nanotubes (MWCNTs) are around $50/g [54,56]. These prices render such nanomaterials economically unviable for large-scale applications requiring square meters of electrode surface area. In contrast, biochar derived from agricultural residues costs only $1–5/kg and achieves 543 mW/m^2^—approximately 70% of the performance of synthetic nanomaterials (773.9 mW/m^2^) [56]. However, biochar-based electrodes suffer from lower electrical conductivity and less defined surface chemistry, leading to shorter operational lifetimes under real wastewater conditions. The trade-off is clear: higher initial investment in nanomaterials yields better performance but uncertain return on investment, while low-cost biochar reduces capital risk but may require more frequent replacement.

Membrane costs represent another significant capital expense. Ion-exchange membranes (e.g., Nafion) are essential for maintaining pH separation and preventing oxygen diffusion in dual-chamber configurations. These membranes cost $500–1000/m^2^ and are susceptible to biofouling, inorganic precipitation, and mechanical degradation [25]. In pilot studies, membrane replacement has been required every 30–60 days in systems with high suspended solids [52]. Membrane-free single-chamber designs reduce costs but suffer from oxygen intrusion at the anode and substrate loss to aerobic respiration, reducing Coulombic efficiency by 30–50% [5]. The absence of comprehensive techno-economic analyses for integrated MFC biorefineries—systems that co-produce electricity, bioplastics (PHB), biosurfactants, and treated water—leaves a critical knowledge gap regarding minimum profitable scale, payback periods, and competitiveness with conventional treatment technologies [46].

**Technical barriers: internal resistance scaling and biofilm instability.** One of the most fundamental physical limitations to MFC scale-up is the relationship between internal resistance and reactor dimensions. Internal resistance (R_internal_) in an MFC comprises ohmic resistance (solution, electrodes, membrane), charge transfer resistance (anode and cathode), and diffusion resistance. As the linear dimension (L) of a reactor increases, the ohmic resistance due to solution conductivity scales approximately with L/A (where A is electrode cross-sectional area). In practice, when scaling from laboratory (mL, L ~ 0.05 m) to pilot scale (1000 L, L ~ 1 m), the electrode spacing must increase to accommodate larger volumes, leading to a 10- to 20-fold increase in ohmic resistance if the same geometry is maintained [41]. This is why laboratory reactors achieve power densities of hundreds to thousands mW/m^2^, while pilot-scale systems (10–1000 L) typically report one to two orders of magnitude lower performance.

Modular designs with multiple small units connected electrically (series for voltage, parallel for current) offer a potential solution, but they introduce new challenges: (i) hydraulic imbalances between modules lead to uneven organic loading, (ii) electrical connections increase system complexity and failure points, and (iii) voltage reversal in series-connected modules can damage electroactive biofilms [45,52]. No modular system has yet demonstrated stable operation beyond 12 months without significant performance decay.

**Membrane fouling and electrode corrosion** drastically reduce operational lifetime in real industrial environments. Real effluents contain suspended solids, colloids, dissolved organics, and inorganic precipitants (e.g., calcium, magnesium, silica) that accumulate on membrane surfaces. Fouling increases membrane resistance, promotes biofilm formation that blocks ion transport, and ultimately requires chemical cleaning or replacement. In the pilot study by [19], membrane permeability decreased by 50% within 45 days when treating food processing wastewater, requiring offline cleaning with dilute acid and base. Electrode corrosion is particularly problematic for metal-based current collectors and catalysts. Copper and zinc electrodes, while cheap, suffer from anodic dissolution in the presence of chloride and organic acids, releasing metal ions that can be toxic to electrogenic biofilms [44,59]. Even carbon-based electrodes experience surface oxidation and loss of functional groups over extended operation, reducing their catalytic activity for ORR and NADH oxidation.

**Maintenance costs and operational complexity**: Based on the limited pilot data available, a preliminary maintenance budget for a 10 m^3^/day MFC system would include: membrane replacement every 2–3 months ($500–1000/m^2^ × 5–10 m^2^ = $2500–10,000 per event), electrode cleaning every 30–60 days (labor and chemicals: $200–500 per event), and periodic reinoculation or bioaugmentation (e.g., *Geobacter sulfurreducens* culture: $500–1000 per batch) [52,56]. These costs currently exceed the revenue from electricity generation (0.1–1.0 kWh/kg COD removed, valued at $0.05–0.15 per kWh). Therefore, MFCs must be positioned as value-added treatment systems where the primary revenue comes from avoided disposal costs, carbon credits, or co-produced bioproducts (PHB, biosurfactants) rather than from electricity sales alone [46].

**Institutional and standardization gaps:** The lack of standardized protocols for performance reporting—as evidenced by the unit inconsistencies in Table 3 and Table 4—prevents meaningful benchmarking and technology comparison. Critical metrics including Coulombic efficiency, normalized energy recovery (kWh/m^3^ treated or kg COD removed), and long-term stability indices (e.g., performance decay rate per month) are rarely reported. Furthermore, most MFC research occurs in academic settings with limited industrial partnership, resulting in technology development that may not align with real-world constraints [50]. The absence of demonstration-scale projects (≥100 m^3^/day) with systematic techno-economic assessment and open data sharing slows learning and investment. Addressing these barriers requires a coordinated effort among material scientists, microbial ecologists, and process engineers, supported by targeted funding for pilot-scale validation and life-cycle assessment.

### 4.6. Future Research Directions

The present study has identified that, despite significant advances in microbial fuel cell (MFC) technology, its large-scale implementation in real industrial environments remains constrained by a series of technical and economic barriers. The following lines of future research are proposed to address these limitations, structured according to the main challenges identified.

#### 4.6.1. Economic Barriers: Costs and Financial Viability

Development of low-cost electrode materials with high performance. One of the principal economic barriers is the high cost of electrodes modified with nanomaterials such as NiO or MWCNTs, which, although they enhance power density, are not viable for large-scale deployment. Future research should prioritize the development of low-cost alternatives, such as biochar derived from residual biomass or recycled carbonaceous materials, while evaluating their long-term performance and environmental footprint across the full life cycle.

**Techno-economic analysis of integrated MFC biorefineries:** The economic feasibility of multiproduct systems that co-generate electricity, bioplastics, and biosurfactants from agro-industrial waste remains largely unexplored. Detailed financial models are needed that account for capital costs (electrodes, membranes, reactors), operating costs (maintenance, substrate supply), and potential revenues from energy generation and value-added products. Such analyses should determine the minimum scale required to achieve profitability and competitiveness with conventional technologies.

**Assessment of environmental externalities and economic incentives:** Research should quantify avoided environmental impacts (e.g., methane emissions, wastewater treatment costs) in monetary terms to make visible the true economic value of MFCs. In parallel, financing mechanisms, carbon credits, and public policies should be explored to facilitate adoption in industrial contexts, particularly within emerging economies.

The African research gap represents a critical oversight given the continent’s acute waste management challenges and energy access deficits. Sub-Saharan Africa generates approximately 180 million tons of municipal solid waste annually, with 70% openly dumped or burned, yet accounts for <2% of MFC publications indexed in Scopus [2]. This disparity reflects broader inequalities in research capacity, laboratory infrastructure, and access to international funding. The absence of Africa-focused MFC research limits technology appropriateness for tropical contexts characterized by high ambient temperatures (25–35 °C), distinct waste composition (higher putrescible fraction, lower paper/plastics), and limited technical infrastructure for system maintenance. Preliminary studies suggest that thermotolerant exoelectrogens (e.g., Thermincola, Thermoanaerobacter) abundant in tropical soils could enable MFC operation at elevated temperatures without cooling, potentially improving kinetics and reducing energy inputs [50]. Context-specific applications with high potential for Africa include: (i) decentralized sanitation-energy systems for off-grid communities combining toilet waste treatment with phone charging and LED lighting, (ii) agro-industrial waste valorization in palm oil, cocoa, and cassava processing where concentrated waste streams create pollution hotspots, and (iii) bioremediation of petroleum-contaminated soils in the Niger Delta and other oil-producing regions [51]. Addressing this research gap requires targeted capacity building, North–South research partnerships with equitable authorship, and funding mechanisms that prioritize locally defined research questions over externally imposed agendas [52]. The African Research Initiative for Bioelectrochemical Systems (ARIBS) proposed by the African Academy of Sciences represents a potential model for coordinated continental effort.

#### 4.6.2. Technical Barriers: Scalability and Operational Stability

**Design and optimization of modular MFC systems for industrial scale:** The transition from laboratory reactors (milliliters to liters) to industrial volumes (cubic meters) presents significant challenges in fluid dynamics, electrode distribution, and internal resistance. Future studies should focus on modular configurations that enable progressive scaling, assessing the impact of series/parallel connections on energy performance and the stability of mixed microbial consortia under high organic load conditions.

**Long-term stability of biofilms under real operating conditions**: Most studies report performance under controlled laboratory conditions, overlooking the population dynamics of electrogenic consortia in response to fluctuations in pH, temperature, organic load, and inhibitors typical of industrial effluents. It is essential to investigate the temporal resilience of bioaugmented consortia (e.g., *Geobacter sulfurreducens* or fungal–bacterial synergies) under non-sterile and variable conditions, identifying key operational parameters that ensure process stability.

**Mitigation of membrane fouling and electrode corrosion**: The useful life of ion-exchange membranes and electrodes in real wastewater is limited by biofouling, inorganic precipitation, and corrosion, which drastically increase replacement costs. Future research should explore protective coatings, periodic cleaning strategies, and membrane-free configurations (such as single-chamber MFCs) tailored to specific industrial residues, evaluating their long-term durability.

**Standardization of measurement protocols and performance metrics:** The current dispersion in units and metrics used to report power density (W/m^3^, mW/m^2^, μW/cm^2^) complicates result comparison and the identification of optimal configurations for each type of waste. A unified measurement protocol is needed that considers not only peak power but also Coulombic efficiency, long-term stability, and organic matter removal rate as key indicators of technological maturity.

#### 4.6.3. Integration with Real Industrial Processes

**Pilot-scale validation with real industrial effluents:** A gap persists between laboratory studies using synthetic substrates and validation under real conditions with complex effluents (e.g., furfural, citrus peels, poultry waste). Long-term pilot plants (exceeding six months) should be implemented in collaboration with industry to evaluate technical performance, operational challenges, and actual maintenance costs, generating data to inform scalability models.

**Development of hybrid systems combining MFCs with anaerobic digestion and algal bioreactors:** Integrating MFCs with complementary technologies could overcome individual limitations: anaerobic digestion manages high organic loads, MFCs polish effluents while generating electricity, and algal cathodes capture CO_2_. Research should explore optimal configurations, energy balances, and synergies in the degradation of recalcitrant compounds.

**Analysis of social acceptance and technology transfer:** Successful implementation of MFCs requires understanding perceptions, training needs, and institutional barriers within industrial sectors. Socio-technical studies that identify adoption facilitators, local training strategies, and adapted business models are necessary to ensure that technological advances effectively translate into industrial practice and contribute to the Sustainable Development Goals.

#### 4.6.4. Benchmarking de Tecnologías MFC

Comparative benchmarking with alternative technologies contextualizes MFC performance and identifies niche applications where competitive advantage exists. Anaerobic digestion (AD), the incumbent technology for organic waste treatment, achieves methane yields of 200–500 L/kg VS with energy recovery of 1–2 kWh/kg COD removed, at scales of 100–10,000 m^3^ with capital costs of $300–800/m^3^ [7]. MFCs currently achieve 0.1–1.0 kWh/kg COD removed at laboratory scale, with projected capital costs of $2000–5000/m^3^ for initial commercial systems [41]. This cost disadvantage suggests that MFCs will not displace AD for bulk waste treatment in the near term. However, MFCs offer distinct advantages for specific applications: (i) dilute waste streams (<1000 mg/L COD) where AD is thermodynamically unfavorable, (ii) wastewaters containing toxic or inhibitory compounds that suppress methanogenesis, (iii) applications requiring low-temperature operation where AD stalls, and (iv) scenarios where electricity is more valuable than biogas (remote locations, off-grid applications) [25,42]. The ability to co-produce valuable biochemicals (PHB, biosurfactants) alongside electricity [46] further differentiates MFCs from AD, which produces only biogas and digestate. Direct comparison with microbial electrolysis cells (MECs) reveals complementary strengths: MECs require external voltage input but produce hydrogen (energy carrier) rather than electricity, achieving energy recovery efficiencies of 70–90% compared to 30–50% for MFCs [30,34]. Integration of MFCs with MECs in a single bioelectrochemical platform could enable switchable operation between electricity generation and hydrogen production based on grid demand and hydrogen markets.

Recent pilot-scale advancements (2025–2026) demonstrate accelerating technology readiness. The literature has reported that a 1 m^3^ pilot system treating food processing wastewater in Japan, achieving 320 mW/m^2^ with *Geobacter sulfurreducens* bioaugmentation over 180 days continuous operation—the longest pilot study to date [19]. The system maintained >80% of initial performance despite seasonal temperature variation (18–32 °C) and organic load fluctuations (COD 1200–3800 mg/L), demonstrating robustness relevant to industrial application. The literature has reported that operated a 500 L modular MFC system treating potato starch wastewater in China, achieving 0.22 W/m^2^ with NiO-modified electrodes while reducing COD by 89% and recovering 0.15 kWh/m^3^. The modular design (10 × 50 L units connected hydraulically in series and electrically in parallel) enabled maintenance of individual modules without system shutdown, addressing a key operational concern [45]. The literature has demonstrated a low-cost (14.13 V from 24 series-connected units) [51]. While power density was modest (~50 mW/m^2^), the capital cost of $150/m^3^ (using zinc/copper electrodes and locally sourced materials) suggests a pathway to affordability in resource-limited settings. These pilot studies, while encouraging, also reveal persistent challenges: electrode fouling required periodic cleaning every 30–60 days, membrane replacement was needed in systems with high suspended solids, and power conditioning electronics added significant system cost. Continued piloting with systematic documentation of operational parameters, maintenance requirements, and total cost of ownership is essential to build the evidence base for commercial adoption.

The quantitative benchmarking of MFC technology against two competing bioelectrochemical or biochemical systems: conventional anaerobic digestion (AD) and microbial electrolysis cells (MECs), in Table 5. The comparison is structured around five key parameters: operational scale, energy recovery, capital investment, main product, and technology readiness level (TRL).

Anaerobic digestion (AD) remains the most mature and widely deployed technology for organic waste treatment, operating reliably at scales of 100–10,000 m^3^ with energy recoveries of 1–2 kWh per kg of COD removed. Its capital cost (USD 300–800 per m^3^) is an order of magnitude lower than current MFC projections, primarily due to decades of optimization, standardized reactor designs, and the absence of expensive membranes or precious metal catalysts. AD’s main product, biogas (50–70% methane), can be directly combusted for heat and power or upgraded to biomethane. However, AD performs poorly at low COD concentrations (<1000 mg/L), is sensitive to inhibitors (ammonia, heavy metals, organic solvents), and requires mesophilic temperatures (30–40 °C) to maintain methanogenic activity.

Microbial fuel cells currently operate at much smaller scales (milliliters to a few cubic meters) and exhibit lower energy recovery (0.1–1.0 kWh/kg COD). Their projected capital cost (USD 2000–5000 per m^3^) is significantly higher than AD, mainly due to ion-exchange membranes, high-surface-area carbon electrodes, and (in some designs) nanomaterial coatings. However, MFCs offer unique advantages: (i) direct electricity generation without gas handling or combustion, (ii) tolerance to low-strength and toxic wastewaters, (iii) ambient temperature operation (15–35 °C), and (iv) the potential to co-produce value-added chemicals such as polyhydroxybutyrate (PHB) or biosurfactants. The technology is currently at TRL 4–5, meaning validated in laboratory and pilot environments but not yet deployed at commercial scale.

Microbial electrolysis cells represent an intermediate option. They require a small external voltage input (0.2–0.8 V) to overcome the thermodynamic barrier for hydrogen evolution, but achieve higher net energy recovery (0.7–0.9 kWh-equivalent per kg COD) when hydrogen is valued as an energy carrier. MECs share similar material costs as MFCs but can avoid membranes in some configurations, slightly reducing capital expenditure (USD 1500–4000 per m^3^). Their main product (hydrogen) is versatile for fuel cells, chemical synthesis, or upgrading to methane. MECs are at TRL 4–6, with several pilot-scale demonstrations (up to 10 m^3^) but not full commercial plants.

Implications for technology selection: No single technology dominates all applications. AD is the clear choice for large-scale, high-strength organic waste treatment (e.g., municipal sludge, food processing effluent) where biogas can be efficiently utilized. MFCs are better suited for niche applications: (a) decentralized treatment of dilute waste streams (<1000 mg/L COD), (b) sites where electricity is more valuable than gas (remote off-grid locations), (c) wastewaters containing toxic compounds that inhibit methanogens, and (d) integrated biorefineries where co-production of bioplastics or biosurfactants generates additional revenue. MECs may become competitive where hydrogen infrastructure exists or where electricity is cheap and hydrogen is expensive. The choice should be guided by local energy prices, waste composition, scale, and downstream valorization pathways.

## 5. Conclusions

The analysis revealed that research on Microbial Fuel Cells and biomass valorization is currently in an exponential growth phase (*R*^2^ = 0.99954), consolidating itself as a multidisciplinary field with a clear predominance of Environmental Sciences (23%) and Chemical Engineering (15%). The geographic distribution of the most highly cited documents highlights the initial leadership of the United States, followed by a progressive decentralization toward emerging economies such as China, India, and Malaysia, albeit with limited explicit international collaboration in the foundational works. With respect to the bioelectrical conversion potential of different residual biomasses, industrial and plant-derived wastes attract the greatest scientific attention and are consistently rated as having high potential. Studies on industrial wastewater and lignocellulosic residues—such as cellulose—demonstrate outstanding yields, although significant dispersion persists toward fundamental research using model substrates, which hinders standardized comparison across biomass types.

Regarding microbial interactions, mixed and synthetic consortia far outperform pure cultures in terms of metabolic versatility and energy efficiency. Fungal–bacterial synergy (e.g., *Trichoderma* sp. with native bacteria) enables the degradation of recalcitrant pollutants such as plastics while generating 0.479 V, whereas bioaugmentation with *Geobacter sulfurreducens* achieves exceptional power densities of 1760 mW/m^2^. Direct interspecies electron transfer (DIET) in engineered consortia further facilitates the co-production of bioplastics and biosurfactants, aligning with circular economy principles. Electrode modification with nanomaterials such as NiO or MWCNTs substantially enhances charge transfer and power density, although metals such as zinc and copper—associated with low-cost configurations—exhibit inferior performance. This trend suggests that nanostructuring represents a priority pathway for improving the technical viability of these systems. Finally, the principal barriers to industrial scalability include biofilm instability, the high cost of modified electrodes, the lack of standardized metrics, and the limited focus on comprehensive life-cycle sustainability. Likewise, the emerging role of MFCs in the field of polymers has been highlighted in this review, both in the controlled degradation of lignocellulosic and plastic waste and in the electro-microbial synthesis of biopolymers (PHB). MFCs act as a true “bridge technology” for polymer engineering: on the one hand, they enable the electrochemical study of enzymatic depolymerization of natural macromolecules (cellulose, lignin) and synthetic plastics; on the other hand, they provide a platform for the bio-electrosynthesis of high-value polymers, such as polyhydroxybutyrate, from the same waste streams. This dual capability—degradation and synthesis—is unique among conventional waste treatment technologies. This dual function positions MFCs as a key technological platform for polymer waste valorization, contributing to closing the materials cycle and reducing plastic pollution, in line with the principles of the circular economy and the protection of terrestrial ecosystems (SDG 15). Therefore, future research in polymer science should integrate MFC-based systems not only as disposal routes but as active components of a circular polymer economy, where end-of-life plastics and lignocellulosic residues become feedstocks for new materials.

Further investigation is required into the temporal stability of mixed microbial consortia under operational fluctuations typical of industrial environments, as well as into optimized fungal–bacterial bioaugmentation configurations for the degradation of recalcitrant wastes such as plastics. Finally, the technical and economic feasibility of integrated biorefineries coupled with MFCs must be analyzed. These systems could co-produce electricity, bioplastics, and biosurfactants from agro-industrial residues. Their limited exploration to date constrains industrial scalability and delays the transition toward an effective circular economy.

## Figures and Tables

**Table 5:** Benchmarking MFC vs. Anaerobic Digestion (AD) and Microbial Electrolysis Cells.

Technology	Scale (m^3^)	Energy Recovery (kWh/kg COD Removed)	Capital Cost (USD/m^3^)	Main Product	TRL
Anaerobic Digestion (AD)	100–10,000	1.0–2.0	300–800	Biogas (CH_4_)	TRL 9
Microbial Fuel Cell (MFC)	0.001–1 (lab/pilot)	0.1–1.0	2000–5000 *	Electricity (direct)	TRL 4–5
Microbial Electrolysis Cell (MEC)	0.001–10	0.7–0.9 (net after external input)	1500–4000	Hydrogen (H_2_)	TRL 4–6

* The quantitative benchmarking of MFC technology against two competing bioelectrochemical or biochemical systems: conventional anaerobic digestion (AD) and microbial electrolysis cells (MECs), in Table 5.

## Data Availability

No new data were created or analyzed in this study.
